# Brain network alterations in autoimmune diseases: Current status and future prospects

**DOI:** 10.1016/j.isci.2026.116682

**Published:** 2026-07-16

**Authors:** Xin Ning Lee, Haiyang Zhang, Huifang Zhou

**Affiliations:** 1 State Key Laboratory of Eye Health, Department of Ophthalmology, Shanghai Ninth People’s Hospital, Shanghai Jiao Tong University School of Medicine, Shanghai, China

**Keywords:** autoimmune disease, brain network, neuroimaging, graph theory

## Abstract

Autoimmune diseases are frequently associated with neurological and cognitive dysfunction. Advances in neuroimaging and graph theory-based connectomics have enabled investigation of large-scale brain network alterations across autoimmune conditions. This review synthesizes structural and functional network studies in representative diseases, including multiple sclerosis, neuromyelitis optica spectrum disorder, systemic lupus erythematosus, thyroid eye disease, type 1 diabetes mellitus, and inflammatory bowel disease. Across these disorders, studies commonly report reduced global and local efficiency, altered modular organization, and regional hub vulnerability, although findings vary across disease subtype, imaging modality, and analytic methodology. Network alterations are often associated with cognitive impairment and neuropsychiatric symptoms, suggesting potential relevance for understanding disease burden and progression. However, current evidence remains heterogeneous and is derived largely from cross-sectional studies with modest sample sizes. Future longitudinal and standardized multimodal studies are needed to clarify the biological and clinical significance of connectomic alterations in autoimmune diseases.

## Graphical abstract

**Figure:**
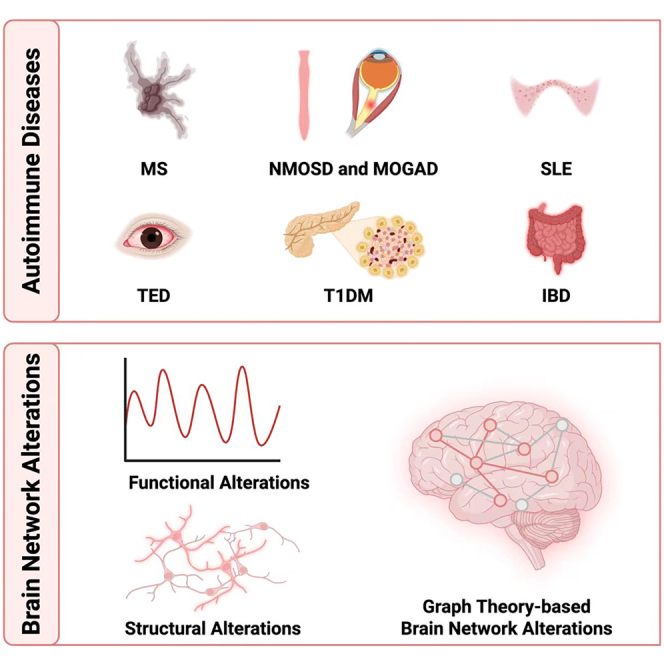

## Teaser

Neuroscience; immunology

## Introduction

Autoimmune diseases represent a complex group of disorders with a significant global impact, affecting approximately 12.5% of the population worldwide and particularly prevalent among women of reproductive age.^1^ These conditions are marked by immune system dysregulation, characterized by polyclonal activation, defects in B or T lymphocyte selection, and altered lymphocyte responses. This immune disturbance results in the presence of autoreactive T and B lymphocytes and autoantibodies, leading to attacks on the body’s own tissues and organs.^2^^,^^3^ Over 100 autoimmune diseases are currently identified, typically classified into organ-specific (e.g., Hashimoto’s thyroiditis, type 1 diabetes mellitus (T1DM)) and systemic (e.g., systemic lupus erythematosus (SLE), rheumatoid arthritis (RA) categories.^4^

Growing evidence suggests that both central and peripheral autoimmune diseases may contribute to neurodegenerative changes, manifesting as cognitive decline and neuropsychiatric disorders.^5^^,^^6^^,^^7^^,^^8^^,^^9^ This occurs as immune dysregulation extends its effects to the central nervous system (CNS), promoting neuroinflammatory responses that disrupt cellular integrity and connectivity in brain regions associated with cognitive and emotional functions.^10^ Although the precise mechanisms behind these changes remain uncertain, investigating brain structure and function offers a path to uncovering shared pathways and potential markers of disease progression.

In recent years, advanced neuroimaging technique such as magnetic resonance imaging (MRI) and magnetoencephalography (MEG), coupled with innovative analysis methods, have enabled detailed characterization of brain changes in autoimmune diseases.^11^^,^^12^^,^^13^^,^^14^^,^^15^ Among these, graph theory, a mathematical framework for studying complex networks, has gained attention for its utility in brain science research. Applying graph theory to study brain networks in autoimmune diseases allows researchers to evaluate structural and functional disruptions in brain connectivity that may reflect disease effects on brain organization.^16^ Studies have increasingly used this approach to identify altered network topologies in autoimmune patients, revealing both unique and shared disruptions across different conditions.

Despite these advances, several challenges limit the interpretability and clinical translation of current findings. Substantial heterogeneity in imaging protocols, preprocessing pipelines, network construction strategies, and patient populations complicates cross-study comparisons. In addition, most available evidence is derived from cross-sectional studies with modest sample sizes, restricting causal inference and longitudinal interpretation. The present review synthesizes graph-theoretical connectomics studies across multiple autoimmune diseases, emphasizing network topology, integration-segregation balance, and hub vulnerability as shared organizational principles. By comparing structural and functional network alterations across immune phenotypes, this review aims to move beyond regional descriptions toward system-level pathophysiological models, while providing an integrative synthesis of current evidence and highlighting methodological considerations, knowledge gaps, and future directions for network-based research in autoimmune conditions.

## Literature search and scope of the review

Relevant literature was identified through systematic searches of PubMed databases using combinations of keywords related to autoimmunity diseases and network-based neuroimaging analysis. Representative autoimmune diseases covered in this review included multiple sclerosis (MS), neuromyelitis optica spectrum disorder (NMOSD), and myelin oligodendrocyte glycoprotein antibody-associated disease (MOGAD), SLE, thyroid eye disease (TED), T1DM, and inflammatory bowel disease (IBD).

This review primarily focused on studies employing graph-theoretical or connectomics-based analyses of structural and functional neuroimaging data. Priority was given to studies reporting network topology metrics, including global efficiency, clustering coefficient, path length, centrality, and modularity.

## Basic concepts of brain networks and graph theory

Brain networks conceptualize the brain as a complex network system composed of numerous neurons or brain regions, where each neuron or brain region is represented as a node, and their interactions are represented as edges.^16^^,^^17^^,^^18^ This topological structure reveals the interconnections and collaborative dynamics among different areas of the brain, while the dynamic changes in the network reflect the brain’s immediacy in function and information processing. Graph theory serves as a crucial mathematical tool for describing and analyzing network features within brain network analysis. Specifically, the construction of brain networks can be approached from anatomical or physiological perspectives, resulting in the formation of structural and functional networks.^19^

**Figure 1:**
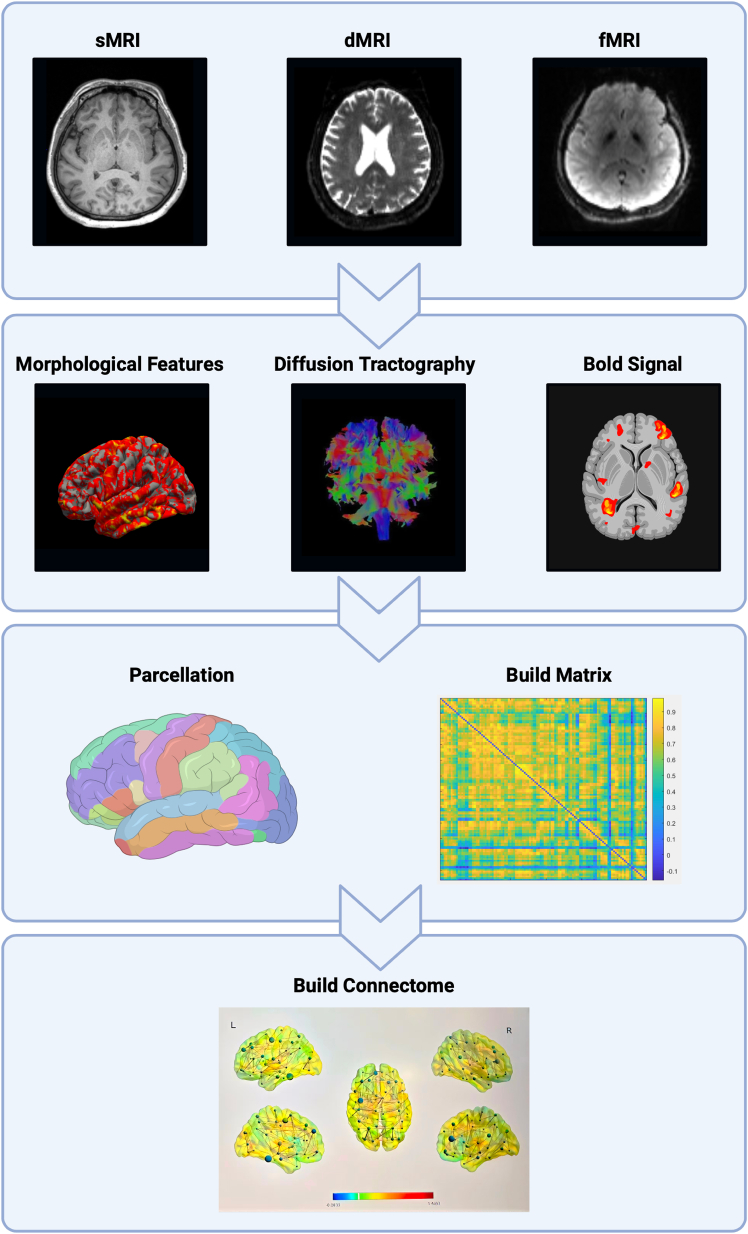
A flowchart for constructing brain networks The construction of human brain networks relies on structural MRI (sMRI), diffusion MRI (dMRI), and functional MRI (fMRI) data and typically involves the following steps: first, network nodes are defined by parcellating the whole brain using an *a priori* atlas. Next, network edges are established by estimating the relationships between these nodes—morphological connectivity is defined by the similarity of morphological features between regions, anatomical connectivity is derived from diffusion tractography, and functional connectivity is based on statistical correlations of BOLD signals across regions. A connectivity matrix is then generated to construct morphological, anatomical, and functional brain networks. BOLD, blood-oxygen-level-dependent; dMRI, diffusion magnetic resonance imaging; fMRI, functional magnetic resonance imaging; sMRI, structural magnetic resonance imaging.

Structural connectome are typically constructed using structural MRI (sMRI) and diffusion MRI (dMRI), which can elucidate anatomical connections in the brain by illustrating the trajectories and strengths of neural fibers. In contrast, functional connectome are established through techniques such as electroencephalography (EEG), MEG, and functional MRI (fMRI), focusing on the temporal correlation of activities between brain regions.^20^ Through complex network analysis based on graph theory, researchers can uncover topological characteristics of brain networks, including metrics such as node degree, clustering coefficient, path length, centrality, and modularity. These metrics facilitate an understanding of how the brain is organized and functions during specific cognitive tasks. Figure 1 shows a flowchart for constructing brain networks.

Graph theory analysis methods primarily encompass functional connectivity (FC) analysis and effective connectivity analysis.^21^^,^^22^ FC analysis examines the statistical dependencies between brain regions, such as the correlation of blood-oxygen-level-dependent (BOLD) signals observed through fMRI data, enabling the identification of dynamic interactions during resting states or specific tasks. Effective connectivity analysis, on the other hand, focuses on causal interactions between brain regions, employing techniques such as dynamic causal modeling (DCM) and Bayesian networks to elucidate how brain regions influence one another and their roles in cognitive processes. Furthermore, calculating global and nodal network metrics can reveal the structural and functional properties of brain networks (Table 1).

**Table 1:** Summary of commonly used global and nodal network properties

Network properties	Description
Global network properties	

Clustering coefficient (Cp)	reflects the average probability that a node’s neighbors are connected, indicating local clustering within the network
Normalized clustering coefficient (γ)	measures the deviation of the network’s clustering coefficient from that of a comparable random network, indicating clustering strength relative to randomness
Characteristic path length (Lp)	represents the average shortest path length between all pairs of nodes, measuring overall network distance
Normalized characteristic path length (λ)	indicates how much the network’s characteristic path length deviates from that of a random network, highlighting efficiency differences
Small-worldness (σ)	evaluates whether the network exhibits “small-world” properties—high clustering combined with short path lengths, supporting efficient communication
Global efficiency (Eg)	quantifies the efficiency of information transfer across the network, indicating the network’s capacity for parallel processing
Local efficiency (Eloc)	measures the network’s robustness and fault tolerance by assessing the efficiency of information transfer within local neighborhoods

Nodal network properties	
Betweenness	measures the extent to which a node lies on the shortest paths between other nodes, indicating its role as an intermediary in network communication
Nodal degree	counts the number of direct connections a node has to other nodes, indicating its immediate connectivity
Betweenness centrality (Bc)	quantifies a node’s centrality by determining the proportion of shortest paths passing through it, highlighting its importance in network flow
Degree centrality (Dc)	assesses centrality based on the node’s degree, or number of direct connections, reflecting its influence within the network
Eigenvector centrality (Ec)	reflects a node’s influence by measuring its connection strength to other highly connected nodes, indicating centrality within significant clusters
Nodal local efficiency (NLe)	evaluates the efficiency of information transfer among a node’s neighbors if the node itself were removed, indicating local network resilience
Nodal efficiency (Ne)	measures the efficiency of parallel information processing associated with a node, reflecting its functional integration within the network
Nodal shortest path length (NLp)	denotes the shortest path length from a given node to any other node in the network, illustrating optimal routing efficiency
Nodal clustering coefficient (NCp)	indicates the degree of clustering around a node, showing how connected its neighbors are to each other

Human brain networks exhibit a unique small-world property, where neurons communicate rapidly through dense short connections within local regions while utilizing fewer long connections for communication between different brain areas.^23^^,^^24^ This structure imparts a high clustering coefficient and moderate characteristic path length to the brain, thereby facilitating efficient information transmission. This property is vital for the complexity and flexibility of information processing, ensuring that the brain achieves a balance between local tasks and global information integration. Additionally, brain networks display modular characteristics, allowing for efficient collaboration among different functional areas.^25^ Connections within modules tend to be denser than those between modules, enhancing the specialization and efficiency of specific functions.

In pathological states, the connection patterns of brain networks can change, which may be associated with alterations in the structure and FC of brain networks. These changes not only affect the efficiency of information processing but may also profoundly impact the cognitive abilities and behaviors of patients. Through graph theory analysis, researchers can identify and quantify these alterations, providing new perspectives for the diagnosis and treatment of these conditions. Moreover, calculating global and nodal network properties aids in revealing the structural and functional characteristics of brain networks, further advancing our understanding of the mechanisms underlying brain function.

## Applications of brain network analysis in autoimmune diseases

### MS

MS is an inflammatory, demyelinating autoimmune disease of the CNS that is commonly associated with impairments in motor, sensory, visual, and cognitive functions.^26^ For most MS patients, the initial episode presents as acute or subacute optic neuritis, brainstem syndromes, or spinal cord lesions, collectively referred to as clinically isolated syndrome (CIS).^27^ A hallmark of MS pathology is white matter damage, marked by axonal loss, demyelination, and disrupted fiber coherence, which drives progressive neurological dysfunction.^28^ While MS has long been recognized as an autoimmune disorder, its neurocognitive consequences and network disorganization are increasingly understood as downstream effects of chronic immune-mediated injury. Over the past three decades, advanced neuroimaging has revealed that lesion burden and volumetric changes on MRI correlate strongly with cognitive performance, offering critical insights into the structural basis of cognitive decline in MS.^29^

**Figure 2:**
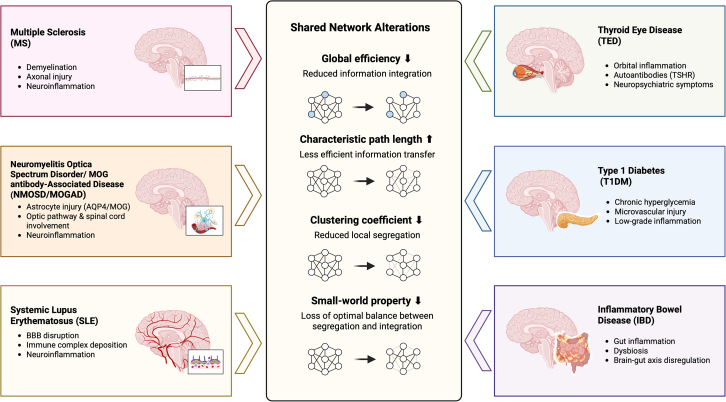
Shared brain network alterations across autoimmune diseases The figure summarizes the shared connectomic alterations observed across autoimmune diseases. Despite distinct immune mechanisms, many disorders demonstrate convergent patterns of reduced network efficiency and compensatory reorganization, suggesting common principles of large-scale brain network disruption. BBB, blood-brain barrier.

Current research (Table 2) indicates that brain networks in MS patients retain small-world properties in both structural and functional networks.^30^^,^^31^ However, structural networks in MS have been reported to show reduced global efficiency, local efficiency, and clustering coefficient, alongside increased characteristic path length, indicating impaired information transfer^31^^,^^38^(Figure 2). These changes emerge early in CIS, where decreased inter-module efficiency between sensorimotor (SMN), default mode (DMN), and fronto-parietal networks precedes overt disability.^37^

**Table 2:** Characteristics and main findings of graph theory studies of MS

Study	Country	Population	Age (Years)	Gender (F/M)	Toolbox	Network size	Modality	Global properties	Local properties	Modularity/Hub	Main finding
He et al.^30^	Canada/US	330 RRMS	38.35(22.2–48.1)	153/177	–	54 ROI	T1W1	Cp. Lp, Eglob, Eloc	Snod, Ne	–	increased white matter lesion load in MS leads to significant decreases in brain network efficiency, particularly in the insula, precentral gyrus, and association areas, reflecting disruptions in large-scale brain connectivity
Shu et al.^31^	China	39 MS 39 HC	37.1 ± 10.7 34.4 ± 9.9	27/12 27/12	SPM8 FSL	90 ROIs	DTI	Sp, Eglob, Eloc	Ne	–	MS reduces brain network efficiency, particularly in the sensorimotor, visual, default-mode, and language systems, with these changes correlating with clinical characteristics
Gamboa et al.^32^	Germany	16 MS 20 HC	35.3 ± 8.3 29.9 ± 7.0	11/5 10/10	SPM8 brain connectivity toolbox	116 ROIs	rs-fMRI	–	–	M	cognitively preserved early MS patients exhibit higher brain modularity at rest, which negatively correlates with dual-task performance, suggesting reduced cognitive compensation capacity and potential early cognitive decline
Van Schependom et al.^33^	Belgium	128 CI 180 CP	55 ± 12 49 ± 11	87/41 102/78	SPM8	17 ROIs	EEG	Lp, σ	Sedge, Dnod, NCp	M	cognitive impairment in MS is linked to neural disconnections, particularly in white matter tracts between hemispheres, leading to widespread network differences
Rocca et al.^34^	Italy	246 MS 55 HC	42.3 (19–60) 41.7 (20–60)	161/85 26/19	MATLAB	116 ROIs	rs-fMRI	Cp, Lp, Dnet, Eglob, Ass, Hrchy	Dnod, BC	H	MS patients exhibit abnormal global network properties and regional hub redistribution, including loss of hubs in key brain regions and altered lateralization, which contribute to cognitive impairment and phenotypic variability by disrupting global integration and information exchange
Shu et al.^35^	China	41 CIS 32 MS 35 HC	35.7 ± 10.7 34.8 ± 8.3 35.0 ± 11.5	26/15 24/8 23/12	FSL SPM8 GRETNA	90 ROIs	DTI, rs-fMRI	Sp, Eglob, Eloc, Lp, Cp, λ, γ, σ	Ne	–	structural connectome disruption occurs as early as the CIS stage, while functional changes emerge later in MS, with both disruptions locally correlated in the sensorimotor component and linked to clinical variables
Liu et al.^36^	China	34 MS 34 CIS 36 HC	35 (21–53) 32 (18–59) 30 (21–59)	28/6 24/10 28/8	FSL FreeSurfer MRtrix3 SPM12	90 ROIs	rs-fMRI T1WI	Eglob, Eloc, λ, γ	Ne	–	disrupted brain network organization appears as early as the CIS stage, with decreased whole-brain and regional efficiency, impaired functional connectivity, and correlations with disease duration
Liu et al.^37^	China	41 CIS 32 MS 35 HC	35.7 ± 10.7 34.8 ± 8.3 35.0 ± 11.5	26/15 24/8 23/12	FSL DPARSF GRETNA	90 ROIs	DTI rs-fMRI	–	–	M	CIS patients exhibit early structural network changes between modules without functional alterations, while MS patients show widespread structural disruptions and impaired inter-module functional efficiency, which are linked to cognitive impairment and physical disability
Charalambous et al.^38^	UK	122 MS 51 HC	48 ± 11 41 ± 13	25/26 36/86	FSL MRtrix3 TractoR	120 ROIs	DWI	ED, Eglob, Eloc, Cp	–	–	structural topological changes in MS, including reduced network efficiency and Cp, are linked to disease progression, with network metrics providing clinically relevant insights into disability and information processing dysfunction beyond traditional measures
Fleischer et al.^39^	Germany	92 MS 101 HC	32.9 ± 9.9 19.7 ± 0.9	65/27 59/42	brain connectivity toolbox	90 ROIs	T1W1	Cp, Eloc, Transitivity	–	M	early RRMS patients exhibit gray matter network reorganization with increased local network properties, suggesting an adaptive cortical response to neuroinflammation that helps maintain brain function independently of disease activity and detectable atrophy
Savini et al.^40^	Italy	68 MS 22 HC	46.7 ± 11.0 36.5 ± 10.8	44/24 12/10	FSL, MRtrix, BCT	10 ROIs grouped into 3 networks	rs-fMRI DTI	Eglob	–	–	in CIMS, cognitive performance is strongly correlated with Eglob, particularly in the DMN and its connections to the cerebellum, whereas in CPMS, brain parenchymal fraction and disability status are more predictive, highlighting the role of cerebellar-DMN connectivity in information processing speed decline in MS
Silemek et al.^41^	Germany	33 RRMS 29 HC	40.9 ± 9.7 41.0 ± 8.5	20/13 19/10	FSL freesurfer MRtrix3 SPM12	80 ROIs	rs-fMRI DTI T1WI	Sp, Cp, Lp	Snod, Dnod	–	cognitive dysfunction in RRMS is associated with structural connectivity loss, while increased functional connectivity may reflect both adaptive and maladaptive mechanisms, highlighting distinct roles of structural and functional networks in cognitive impairment
Welton et al.^42^	UK	37 MS 23 HC	48 ± 11 42 ± 12	30/7 17/6	FSL freesurfer FMRIB’s diffusion toolbox	164 ROIs	DTI rs-fMRI	Cp, Lp, Eglob, σ	–	M	cognitive impairment in MS is linked to increased network segregation and reduced integration, with network disruption emerging as a key determinant of cognitive deficits, and graph-based metrics showing reliability over a one-month period, supporting their potential as surrogate outcomes in clinical trials
Riazi et al.^43^	Germany	12 MS 12 HC	–	5/7 8/4	GIFT SPM8	37 ROIs	rs-fMRI	Sp, Cp, Eglob	–	–	spectral ICA provides more reliable brain connectivity components and improved dynamic state selection, revealing significantly decreased anterior cingulate connectivity in MS patients, along with weaker core but stronger peripheral connectivity in the posterior cingulate cortex
van der Weijden et al.^44^	Brazil	30 RRMS 19 PMS 19 HC	35.7 ± 7.6 49.3 ± 8.9 41.3 ± 12.8	21/9 11/8 15/4	SPM12 BRAPH LST	116 ROIs	T1W1	Dnet, Sp, Lp, Eglob, Eloc, Cp, transtivity, Ass, σ	NLp, Dnod, Snod, CC, Eglob, Eloc, Participation, Within module *Z* score	M	lesion filling in T1W1 enhances detection of network alterations in MS by reducing variability across subjects, but it also introduces significant artifacts, particularly in individuals with higher lesion loads, and should be applied cautiously
Abdolalizadeh et al.^45^	Iran	60 RRMS 26 HC	30.75 ± 7.07 30.65 ± 7.82	49/11 22/4	MRtrix3 FSL ANTs FreeSurfer	120 ROIs	DWI	Eglob	–	M	MS subjects exhibit higher modularity and lower Eglob, with increased modularity being associated with cognitive decline and T2 lesion load, suggesting that disrupted intermodular connections due to lesions contribute to cognitive impairment without preserving brain function
Hamwi et al.^46^	Canada	24 MS	26 (18–38)	14/10	brain connectivity toolbox	not stated	rs-fMRI	Dnet, ED, aBC, Cp, Lp, γ, λ, σ	–	–	both cross-sectionally and longitudinally network parameters serve as useful markers of disease severity in MS, with changes in these parameters correlating with neuronal injury markers and reflecting cortical changes relevant to progressive nonrelapsing MS
Has Silemek et al.^47^	Germany	37 RRMS 39 HC	42.2 ± 9.5 42.2 ± 8.3	22/15 22/17	MRtrix3 SPM12 brainwaver igraph tnet	160 ROIs	DTI rs-fMRI MEG	–	Snod, Dnod	H	while structural connectivity declined over time in RRMS, hub connectivity in the default-mode network (DMN) was preserved, suggesting a resilience mechanism that may mimic physiological reorganization seen in healthy aging, with lower structural and functional connectivity in the DMN being linked to poorer cognitive performance in attention and memory
von Schwanenflug et al.^48^	Germany	75 MS 75 HC	42.0 ± 11.0 40.2 ± 11.8	45/30 45/30	network community toolbox	274 ROIs	rs-fMRI	flexibility, promiscuity, cohesion, disjointedness and entropy	–	–	patients with MS show a hyperflexible reorganization of brain activity, particularly in pericentral, subcortical, and limbic regions, with increased global flexibility, promiscuity, entropy, and cohesion, and these changes correlate with clinical disability, suggesting that alterations in multilayer temporal dynamics contribute to MS manifestation
Fleischer et al.^49^	multiple countries	406 MS 153 HC	35.7 ± 9.1 35.0 ± 10.1	280/126 96/57	brain connectivity toolbox	average 6977 ROIs	T1W1	Dnet, Eglob, transitivity	–	–	early gray matter network reorganization, characterized by lower Dnet and Eglob, predicts disability accumulation in RRMS patients, providing more relevant information than conventional MRI measures such as gray matter atrophy and white matter lesion load in predicting EDSS progression over 5 years

MS, multiple sclerosis; RRMS, relapsing-remitting MS; PMS, progressive MS; CIS, clinically isolated syndrome; HC, heathy controls; ROIs, regions of interest; DTI, diffusion tensor imaging; DWI, diffusion-weighted image; rs-fMRI, resting-state functional magnetic resonance imaging; TIWI, T1-weighted image; MEG, magnetoencephalography; Sp, network strength; ED, edge density; Eglob, global efficiency; Eloc, local efficiency; Cp, clustering coefficient; Lp, shortest path length; γ, normalized clustering coefficient; λ, normalized shortest path length; σ, small-world parameters; Ass, assortavity; Ne, nodal efficiency; Snod, nodal strength; BC, betweeness centrality; DC, degree centality; NCp, clustering coefficient; Dnod, nodal degree; NLe, local efficiency; NPL, node shortest path.

Functional networks in MS tend to show hypo-connectivity in the SMN, DMN, and visual network, with reduced local efficiency and clustering coefficient correlating with cognitive deficits.^34^^,^^42^ In contrast, CIS patients show preserved global functional topology but exhibit early nodal inefficiency in the temporal and occipital lobes, suggesting selective vulnerability of visual and DMN hubs.^36^ EEG-based analyses further reveal reduced theta/delta coherence and altered small-worldness in cognitively impaired patients, suggesting an association between neural disconnection patterns and cognitive decline.^33^ Dynamic FC analyses reveal hyperflexible reorganization in MS, characterized by increased promiscuity and entropy in pericentral and limbic regions, which correlates with clinical disability.^48^

MS is associated with disrupted nodal efficiency in critical hubs, including the superior frontal gyrus, precuneus, and insula, impairing cognitive and SMN integration.^36^^,^^37^ Subcortical structures like the thalamus and caudate show altered connectivity patterns, with thalamic characteristic path length increases correlating with Expanded Disability Status Scale (EDSS) scores.^31^ The cerebellum’s integration into the DMN further deteriorates in cognitively impaired patients, exacerbating processing speed deficits.^40^ Early MS features adaptive network reorganization, such as increased modularity and local efficiency, to offset damage.^32^^,^^39^ However, excessive modularity in advanced stages reflects dysfunctional segregation, potentially disrupting inter-network communication and accelerating cognitive decline.^45^ Similarly, functional hyperconnectivity within the DMN may initially compensate for structural loss but ultimately contributes to inefficient processing^41^

Graph theory-based network metrics offer transformative potential as prognostic and diagnostic tools in MS. Reduced global efficiency and network degree in structural networks have been shown to predict 5-year disability progression at the group level, in some studies outperforming conventional MRI measures such as lesion load or atrophy, highlighting their utility for early risk stratification.^49^ Furthermore, increased modularity and aberrant dynamic flexibility in functional networks correlate with cognitive deficits and higher EDSS scores, providing quantifiable associations between network disorganization and clinical disability.^42^^,^^48^ Notably, structural-functional decoupling within the DMN emerges as a sensitive biomarker of early neurodegeneration processes, potentially preceding overt cognitive decline.^47^ Post-treatment studies link improved network metrics to reduced neuronal injury biomarkers (N-Acetylaspartate-to-creatine ratio (NAA/Cr), serum neurofilament light chain (sNfL)), underscoring their utility in monitoring therapeutic efficacy.^46^ Methodologically, lesion filling enhances sensitivity to network alterations in MS but introduces artifacts in high lesion-load patients, necessitating cautious application.^44^

These findings underscore the potential clinical relevance of network analysis for identifying patients at risk of rapid progression, supporting disease stratification and longitudinal monitoring. By capturing both adaptive plasticity and dysfunctional reorganization, graph theory provides a systems-level framework linking neuroimaging findings with clinical phenotypes in MS. Moving forward, integrating immunological markers with brain network metrics may offer novel insights into how autoimmune processes reshape brain connectivity. Understanding the interplay between autoimmunity and neural network reorganization could refine disease stratification and promote precision medicine in MS.

### NMOSD and MOGAD

NMOSD and MOGAD are acute inflammatory disorders that mainly affect the optic nerves and spinal cord. Both conditions share clinical features such as acute optic neuritis, transverse myelitis, and brainstem syndromes.^50^ However, their pathogenesis diverges: NMOSD is driven by pathogenic IgG autoantibodies targeting aquaporin-4 (AQP4-IgG), whereas MOGAD is mediated by autoantibodies against myelin oligodendrocyte glycoprotein (MOG-IgG). Patients with NMOSD often exhibit brain abnormalities, including widespread cortical thinning, deep gray matter atrophy, and abnormal FC.^51^^,^^52^ These changes may reflect the disease-related effects on brain structure and function, subsequently affecting neurological function and quality of life.

Graph theory-based analyses (Table 3) reveal that NMOSD patients retain small-world network properties at the global level, suggesting preserved basic topological.^54^^,^^59^ However, NMOSD is characterized by reductions in global efficiency, local efficiency, and small-worldness, alongside increased characteristic path length compared to healthy controls^57^^,^^59^(Figure 2). In parallel, Ding et al.^63^ demonstrated that MOGAD patients exhibit impaired structural connectivity networks, marked by increased characteristic path length and decreased global efficiency, alongside reduced structural-functional coupling, correlating with clinical disability. These findings indicate a decline in the brain’s capacity for integrated and segregated information processing, despite structural similarities to healthy networks.^57^ The observed alterations in global and local efficiency may reflect downstream effects of autoantibody-mediated demyelination and glial dysfunction on large-scale network organization.

**Table 3:** Characteristics and main findings of graph theory studies of NMOSD and MOGAD

Study	Country	Population	Age (Years)	Gender (F/M)	Toolbox	Network size	Modality	Global properties	Local properties	Modularity/Hub	Main finding
Cho et al.^53^	South Korea	14 NMOSD 21 HC	39 (21–68) 30 (22–56)	13/1 20/1	FSL MATLAB brain connectivity toolbox	90 ROIs	DWI	Sp, ED,Cp, Lp, σ	Dnod, Snod, Eloc, participation coefficients, regional efficiency	–	in NMOSD patients, decreased white matter network strength and disrupted sub-networks were associated with impaired cognitive functions, suggesting that cognitive dysfunction is linked to white matter dysconnectivity, including alterations in the default mode network
Bigaut et al.^54^	France	12 NMOSD 20 HC	45.6 ± 13.6 38.0 ± 8.0	6/6 11/9	SPM8	90 ROIs	rs-fMRI	Eglob, Cp, degree distribution	–	M	in NMOSD patients, while global small-world topology was preserved, decreased connectivity in visual and sensorimotor networks exhibited high variability, with hub disruption correlating with EDSS, suggesting neuronal reorganization and brain plasticity in response to disability
Backner et al.^55^	Germany	23 NMOSD 18 CIS-ON 21 CIS-nON 26 HC	46.7 ± 14.5 31.2 ± 7.7 33.4 ± 8.6 43.7 ± 15.7	20/3 11/7 11/10 22/4	brain voyager brain connectivity toolbox	62 rois	rs-fMRI	Cp, Lp, γ, λ, Eglob, Eloc	Dnod	M	in patients with ON, visual network density was reduced across all groups, with increased connectivity in ON groups particularly in dorsal-lateral regions, while efficiency and modularity were selectively reduced in CIS, highlighting disease-specific cortical reorganization linked to neurologic deficits
Wang et al.^56^	China	19 NMOSD 22 HC	48.58 ± 13.38 47.78 ± 11.63	19/0 22/0	dparsfa gretna	not stated	rs-fMRI	short-range FCD, long range FCD	–	–	patients with NMOSD exhibit widespread brain dysfunction after ON, characterized by altered long- and short-range FCD, with these changes correlating with structural and microvascular alterations around the optic nerve head
Zheng et al.^57^	China	48 NMOSD 50 HC	42 (18–65) 40.5 (21–67)	44/4 42/8	PANDA GRETNA	90 ROIs	DTI	Cp, Lp, γ, λ, σ, Eglob, Eloc	BC, DC, NCp, Ne, NLp	–	NMOSD patients exhibit disrupted white matter networks with decreased Eglob and Eloc, increased Lp, and altered nodal properties, particularly in the default mode and visual systems, which correlate with cognitive impairment and disability severity
Chen et al.^58^	China	51 RRMS 42 NMOSD 56 HC	40.67 ± 14.08 40.48 ± 13.70 37.38 ± 14.29	32/19 39/3 31/25	FSL MRtrix GRETNA	148 ROIs	DTI rs-fMRI	Cp, Lp, Eglob, Eloc	–	–	RRMS and NMOSD patients exhibit distinct patterns of fiber connection damage, with RRMS showing greater reductions in long-range fiber number and FA, which correlate with brain atrophy and structural network efficiency, while short-term follow-up reveals no significant progression in either disease
Cho et al.^59^	South Korea	68 MS 50 NMOSD 26 HC	34.7 ± 8.7 44.4 ± 11.6 35.3 ± 11.3	51/17 42/8 21/5	FSL DTK PANDA BCT	90 ROIs	DTI	Cp, Lp, Eglob, Eloc	Dnod, Snod, NLE	–	both MS and NMOSD exhibit disrupted white matter network organization, but MS shows more extensive network alterations, particularly in thalamic and inferomedial temporal connections, with network dysfunction correlating with disability and disease duration in both conditions
Ma et al.^60^	China	18 NMOSD 22 HC	42.11 ± 12.17 44.36 ± 9.05	16/2 19/3	CAT12 SPM12 DPABI GRETNA	90 ROIs	T1W1 rs-fMRI	σ, Cp, Lp, λ, Eglob, Eloc	Dnod, BC, Ne	–	NMOSD patients with mild disability exhibit compensatory increases in morphological brain network properties, which better correlate with clinical assessments and predict EDSS worsening compared to functional networks
Wang et al.^61^	China	21 NMOSD-CI 17 NMOSD-CP 39 HC	54.38 ± 12.21 36.52 ± 12.63 43.28 ± 14.10	14/7 15/2 27/12	freesurfer BRAPH 2.0	38 ROIs	T1W1	σ, Cp,Lp, Eglob, Eloc, Sp,transitivity, Dnet	NCp, NPl, Eglob, Eloc, DC, BC	M	NMOSD patients with cognitive impairment show abnormal intrinsic hippocampal morphological networks, with nodal metrics of the left hippocampal tail correlating with neurocognitive scores, suggesting their potential as markers for identifying cognitive impairment
Wang et al.^62^	China	30 NMOSD 45 HC	37.70 ± 11.99 41.84 ± 11.23	27/3 33/12	CAT12 GIFT RESTplus GRETNA	43 ROIs grouped into 8 networks	rs-fMRI	Cp, γ, Lp, λ, σ	NCp, DC, Enod	–	NMOSD patients exhibit significant alterations in dynamic rather than static networks, with increased low-connectivity state occurrence and dwell time, fewer state transitions, and disrupted topological organization, which correlate with clinical disability and suggest impaired brain network communication over time
Ding et al.^63^	China	32 MOGAD 30 HC	7.3 ± 2.5 7.6 ± 2.0	20/12 17/13	LST toolbox PANDA DPABI GRETNA	90 ROIs	DTI rs-fMRI	Cp, Lp. Eglob, Eloc, γ, λ, σ	Ncp, BC, Ne, NLe, NCp	–	pediatric MOGAD exhibits significant structural network impairments, SC-FC decoupling, and altered nodal properties, which correlate with clinical disability and demonstrate potential as biomarkers for assessing brain damage

NMOSD, neuromyelitis optica spectrum disorder; NMOSD-CI, cognitively impaired NMOSD; NMOSD-CP, cognitively preserved; MS, multiple sclerosis; RRMS, relapsing-remitting multiple sclerosis; CIS, clinically isolated syndrome; CIS-ON, CIS with optic neuritis; CIS-nON, CIS without optic neuriris; MOGAD, myelin oligodendrocyte glycoprotein antibody-associated disease; HC, heathy controls; ROIs, regions of interest; DTI, diffusion tensor imaging; DWI, diffusion-weighted image; rs-fMRI, resting-state functional magnetic resonance imaging; TIWI, T1-weighted image; MEG, magnetoencephalography; Sp, network strength; ED, edge density; Eglob, global efficiency; Eloc, local efficiency; Cp, clustering coefficient; Lp, shortest path length; γ, normalized clustering coefficient; λ, normalized shortest path length; σ, small-world parameters; Ass, assortavity; Ne, nodal efficiency; Snod, nodal strength; BC, betweeness centrality; DC, degree centality; NCp, clustering coefficient; Dnod, nodal degree; NLe, local efficiency; NPL, node shortest path.

At the regional level, NMOSD patients exhibit disrupted nodal centrality and efficiency in critical brain regions. The left angular gyrus and superior parietal gyrus show reduced short- and long-range FC density, impairing visuospatial and attentional processing.^56^ Key hubs within the DMN and central executive network demonstrate diminished nodal efficiency and degree centrality, reflecting inefficient local processing and inter-regional communication.^53^^,^^57^ Subcortical structures such as the caudate and thalamus display altered connectivity patterns, with thalamic nodal shortest path metrics correlating positively with EDSS scores.^53^ Hippocampal morphological networks are also disrupted in cognitively impaired NMOSD patients, with nodal abnormalities in the left hippocampal tail and CA1-body correlating with neurocognitive scores.^61^ The selective vulnerability of these hubs may reflect region-specific susceptibility to autoimmune-mediated astrocytopathy and inflammatory cytokine exposure, although direct causal mechanisms remain to be established.

Distinct from relapsing-remitting multiple sclerosis (RRMS), NMOSD appears to preferentially disrupt long-range fibers while sparing short-range connections and inferomedial temporal regions, which are severely affected in MS.^58^^,^^59^ Compared to CIS with optic neuritis (CIS-ON), NMOSD-ON patients share reduced visual network density but retain normal efficiency and modularity, suggesting disease-specific compensatory mechanisms.^55^ Unlike MS, which shows pronounced thalamo-temporal and hippocampal disruptions, NMOSD has been associated with relative sparing of these regions, highlighting divergent network vulnerabilities.^59^

Clinically, these topological disruptions are associated with functional impairment. Reduced clustering coefficients in the bilateral anterior cingulate cortex correlate with poorer cognitive performance, while increased path length in thalamic regions associates with higher EDSS scores.^54^^,^^57^ Notably, preserved small-world organization coexists with local network randomization in SMN and visual networks, suggesting compensatory plasticity to mitigate disability.^54^^,^^62^

These findings emphasize that NMOSD and MOGAD, although sharing clinical presentations with MS, exhibit distinct patterns of network reorganization that likely reflect antibody-specific pathophysiological processes. Advanced machine learning models leveraging these topological features demonstrate promising classification and prognostic performance in research settings, highlighting their potential role as candidate biomarkers.^60^^,^^63^

### SLE

SLE is a chronic autoimmune disease that primarily affects women, resulting in multi-system and multi-organ damage.^64^ Due to widespread cerebral vasculitis, SLE patients often experience brain damage and abnormalities.^65^ Recent neuroimaging studies shown that SLE patients showed increased brain activity which commonly affect DMN and the limbic system.^66^ Immune-mediated vascular injury, complement activation, and cytokine imbalance are implicated in cerebral pathology, resulting in neuroinflammation, endothelial dysfunction, and therefore disrupted neural connectivity.

Graph theory-based brain network analysis (Figure 2; Table 4) has revealed that while SLE patients retain small-world network characteristics, both global and local efficiency are significantly reduced compared to healthy controls.^68^ These disruptions are further characterized by increased characteristic path length and decreased clustering coefficient, suggesting possible impairments in global integration and local specialization.^67^^,^^69^ Notably, cognitive decline in SLE patients is associated with increased characteristic path lengths, suggesting a decline in efficient information transfer within the brain.^68^ Furthermore, these alterations correlate with serum complement C4 levels and anti-double-stranded DNA (ADNA) autoantibody activity, suggesting a potential link between immune dysfunction and disrupted brain network topology.^70^^,^^73^ Patients with neuropsychiatric SLE (NPSLE) exhibit more pronounced topological disruptions, particularly in the DMN and SMN, compared to non-NPSLE patients.^72^

**Table 4:** Characteristics and main findings of graph theory studies of SLE, TED, T1DM, and IBD

Study	Country	Population	Age (Years)	Gender (F/M)	Toolbox	Network size	Modality	Global properties	Local properties	Modularity/Hub	Main finding

**SLE**											

Xu et al.^67^	China	29 non-NPSLE 29 HC	29.50 ± 9.91 30.97 ± 9.76	29/0 29/0	GRETNA	90 ROIs	DTI	Sp, Eglob, Eloc, Cp, Lp, γ, λ, σ	Ne, Dnod	–	non-NPSLE patients exhibited impaired Eglob and Eloc, altered hub distribution, and disrupted structural connectivity, correlating with disease activity and suggesting underlying neurocognitive deficits
Wiseman et al.^68^	UK	47 SLE	48.5 (13.7)	43/4	brain connectivity toolbox	85 ROIs	DTI	ED, Sp, Lp, Eglob, Cp	–	H	brain network properties were independently associated with cognitive abilities and systemic damage in SLE, suggesting connectomics as a potential tool for monitoring cognitive function and white matter integrity
Zhao et al.^69^	China	28 non-NPSLE 24 HC	30.5 ± 10.5 29.5 ± 7.7	28/0 24/0	GRETNA	90 ROIs	DTI	Cp, Lp, Eglob, Eloc, γ, λ, σ	Ne, Snod, BC	H	non-NPSLE patients exhibited altered global and nodal network topology, with decreased efficiency and disrupted connectivity in regions related to motor and cognitive functions, alongside structural abnormalities in major white matter tracts, suggesting impaired brain connectivity despite the absence of neuropsychiatric symptoms
Preziosa et al.^70^	Italy	32 SLE 32 HC	39.9 ± 12.4 40.4 ± 12.6	6/26 6/26	brain connectivity toolbox	116 ROIs	DTI rs-fMRI	Sp, Ass, transitivity, Eglob, Lp	Snod, BC, NCp	H	patients with SLE, particularly those with ADNA autoantibodies, exhibited disrupted global and nodal structural connectivity, while functional network integrity remained largely preserved, potentially supporting clinical stability
Cao et al.^71^	China	41 SLE 35 HC	40.6 ± 1.4 38.1 ± 1.3	41/0 35/0	GRETNA	116 ROIs	fMRI	γ, λ, σ, Eloc, Eglob	BC, DC, Ne	–	SLE patients exhibited abnormal nodal network metrics, with decreased efficiency and centrality in key regions, along with disrupted functional connectivity between the basal ganglia and cerebellum, which may contribute to cognitive dysfunction and serve as markers for disease progression
Ao et al.^72^	China	29 NPSLE 21 non-NPSLE 32 HC	30 (23–28) 28 (22–32.5) 33 (24–36.5)	29/0 21/0 32/0	GRETNA	90 ROIs	DTI	Eglob, Eloc, Lp,Cp, σ	DC, BC, Ne, NLe, NCp	–	significant global and local topological alterations were observed in both non-NPSLE and NPSLE patients, with more pronounced changes in NPSLE, particularly in the default mode and sensorimotor networks, correlating with lesion burden and clinical parameters, offering potential as biomarkers for clinical diagnosis
Li et al.^73^	China	18 SLE with MoCa-H 20 SLE with MoCa-L 44 HC	36.2 ± 5.3 49.0 (43.6–52.3) 43.3 ± 1.2	18/0 20/0 44/0	Gretna	90 ROIs	rs-fMRI	Cp, Eglob, Eloc	Ne	–	in SLE patients with low MoCA scores, disrupted functional brain network topological properties, including changes in efficiency and path length, were correlated with clinical markers, suggesting their potential as imaging biomarkers for disease monitoring

**TED**											

Wu et al.^74^	China	27 TED 27 TED	38.5 ± 14.9 38.5 ± 14.5	19/8 19/8	PANDA GRETNA	90 ROIs	DTI	Lp, Cp,σ, Eglob, Eloc	BC, Enod, Dnod	–	patients with TED exhibit disrupted structural brain network connectivity, with altered nodal properties correlating with clinical and neuropsychological dysfunction, suggesting that these network disruptions are associated with the disease’s clinical manifestations
Luo et al.^75^	China	37 aTED 35 iTED 23 HC	43.16 ± 11.84 39.69 ± 14.04 37.22 ± 13.18	22/15 27/8 18/5	SPM8 FSL	90 ROIs	DTI	Cp, Lp, γ, λ, σ, Eglob, Eloc	Ne, BC	–	active TED patients exhibit decreased Eglob and Eloc, as well as reduced Ne in regions such as the orbital superior frontal gyrus, hippocampus, and amygdala, with significant correlations between network properties and anxiety/depression scores, exophthalmos, and intraocular pressure, suggesting altered brain networks linked to emotional and cognitive dysfunction in TED
Zhou et al.^76^	China	28 DON 38 nDON 30 HC	54.00 ± 11.44 49.18 ± 8.41 50.30 ± 11.97	15/13 23/15 19/11	GRETNA	90 ROIs	rs-fMRI	Eglob, Eloc, Cp, Lp, γ,λ, σ	Bc, Dc, NLe, Ne,NLP,NCp	–	DON patients show significant alterations in both static and dynamic brain network properties, including reduced Eglob and increased Lp, with abnormalities in the orbitofrontal cortex and visual network
Fang et al.^77^	China	25 TED 25 HC	54.84 ± 3.22 55.36 ± 4.48	15/10 15/10	GRETNA	160 ROIs	rs-fMRI	Cp, Lp, γ, λ, σ, Eglob, Eloc	Dnod, Ne, BC	–	TED patients exhibit abnormal brain functional network connectivity, with altered BC and Ne, particularly within the DMN, VN, SMN, and CON, offering insights into the neural mechanisms behind visual loss, emotional regulation issues, and cognitive deficits in these patients
Li et al.^78^	China	32 TED 32 HC	58.81 ± 4.53 58.34 ± 3.16	11/21 12/20	GRETNA	128 ROIs	rs-fMRI	Cp, Lp, γ, λ, σ, Eglob, Eloc	Bc, Dc, NCp, Ne, NLe, NLP	–	patients with TAO exhibited altered white matter functional networks, including decreased small-world metrics, reduced betweenness centrality in the splenium, and disrupted modular organization, suggesting impacts on visual and cognitive functions

**T1DM**											

Lyoo et al.^79^	China	81 T1DM 38 HC	32.5 (4.6) 30.8 (5.1)	41/40 19/19	FreeSurfer brain connectivity toolbox	64 ROIs	T1W1	Eloc, Eglob, Lp	–	H	T1DM patients exhibit disrupted prefrontal network organization, leading to impaired top-down cognitive control and reduced integration with language, memory, and emotional processing networks
van Duinkerken,^80^	Netherlands	51 T1DM-PR 53 T1DM-nonPR 49 HC	44.5 ± 7.2 37.8 ± 9.2 36.7 ± 11.2	30/21 33/20 30/19	FSL MATLAB brain connectivity toolbox	90 ROIs	T1W1	Lp, Cp, Dnet, properties size, connectivity density	NCp, NLp, BC	–	global gray matter network topology was more randomly organized in T1DM, with the greatest disruptions in patients with PR, and these alterations were associated with cognitive deficits and reduced fractional anisotropy
van Duinkerken,^81^	Netherlands	104 T1DM 49 HC	41.1 ± 8.9 36.7 ± 11.2	30/19 63/41	FSL fast-ECM	10 ROIs	rs-fMRI	–	ECM, DC	–	patients with T1DM showed reduced ECM and DC in the bilateral thalamus and dorsal striatum, with the lowest values in those without PR, while early connectivity reorganization in these patients was lost with disease progression.

**IBD**											

Liu et al.^82^	China	43 CD 37 HC	31.72 ± 7.93 30.49 ± 5.84	15/28 12/25	CONN functional connectivity toolbox GIFT toolbox	48 ROIs	rs-fMRI	Cp, Eloc, Lp, Eglob	Snod, NCp, NLE	–	CD patients exhibit disrupted local and global topological patterns in brain functional networks, including altered nodal metrics in key networks such as the subcortical, sensorimotor, cognitive control, and default-mode networks, with changes linked to anxiety, depression, and disease duration, providing insights into the neuroimaging mechanisms underlying pain, sensory, emotional, and cognitive processing in CD
Polverino et al.^83^	Italy	25 IBD 28 HC	42.28 ± 13.15 45.18 ± 12.26	10/15 12/16	fieldtrip toolbox	90 ROIs	MEG	the leaf fraction, the tree hierarchy, the diameter, the degree divergence	Dnod, BC	–	the BC of the left hippocampus was higher in patients with UC and CD compared to healthy controls, particularly in the gamma frequency band, suggesting increased involvement in information flow within the brain network due to the inflammatory process

SLE, systemic lupus erythematosus; NPSLE, neuropsychiatric SLE; TED, thyroid eye disease; aTED, active TED; iTED, inactive TED; DON, dysthyroid optic neuropathy; T1DM, type 1 diabetes mellitus; T1DM-PDR, T1DM with proliferative diabetic retinopathy; CD, Crohn’s disease; IBD, inflammatory bowel disease; HC, heathy controls; MoCa, Montreal Cognitive Assessment; ROIs, regions of interest; DTI, diffusion tensor imaging; rs-fMRI, resting-state functional magnetic resonance imaging; TIWI,T1-weighted image; MEG, magnetoencephalography; Sp, network strength; ED, edge density; Eglob, global efficiency; Eloc, local efficiency; Cp, clustering coefficient; Lp, shortest path length; γ, normalized clustering coefficient; λ, normalized shortest path length; σ, small-world parameters; Ass, assortavity; Ne, nodal efficiency; Snod, nodal strength; BC, betweeness centrality; DC, degree centality; NCp, clustering coefficient; Dnod, nodal degree; NLe: local efficiency; NPL, node shortest path; ECM, eigenvector centrality maps.

Examining nodal network properties, several brain regions including the insula, putamen, globus pallidus, Heschl’s gyrus, and anterior cingulate cortex, exhibit reduced nodal efficiency and degree centrality in SLE patients at the group level.^68^^,^^69^^,^^71^^,^^73^ These nodal deficits are correlated with impaired movement control, executive function, and working memory.^69^ FC analysis highlights abnormal connectivity in regions such as the hippocampus and basal ganglia-cerebellar pathways, critical areas for memory and cognitive processing.^71^ Structural connectivity deficits, particularly in non-NPSLE patients, are more pronounced than functional alterations, suggesting early subclinical neural changes.^67^^,^^70^ Key cognitive hubs, such as the putamen and cerebellar vermis, show disrupted structural integrity, further underscoring their role in SLE-related cognitive dysfunction.^71^

These findings highlight the potential of graph theory metrics as biomarkers for assessing disease severity and progression. The hippocampus, though less studied structurally, demonstrates functional disruptions associated with memory dysfunction, suggesting that its nodal metrics could help evaluate memory impairment in SLE.^71^ Advanced classification models leveraging these topological features can even differentiate NPSLE from non-NPSLE with 87% accuracy, emphasizing their clinical utility.^72^ Future studies integrating immune phenotyping with connectomic analysis could clarify how systemic immune dysfunction shapes brain architecture and cognitive outcomes in SLE.

### TED

TED is an organ-specific autoimmune disorder primarily targeting orbital tissues, triggered by aberrant immune responses against thyroid-stimulating hormone receptors (TSHR) and insulin-like growth factor-1 receptors (IGF-1Rs).^84^ It clinical manifestation such as proptosis, eyelid retraction, diplopia, and strabismus reflect both inflammatory and fibrotic changes within the orbit. Some patients may experience dysthyroid optic neuropathy (DON), leading to significant visual impairment.^85^ The clinical course of TED is typically divided into active and inactive phases based on the clinical activity score. In addition to ocular symptoms, TED patients may suffer from emotional and cognitive impairments, such as anxiety, depression, and memory loss.^86^^,^^87^ Beyond ocular pathology, emerging neuroimaging studies reveal TED-associated brain alterations in regions governing vision, cognition, and emotion, such as gray matter volume loss, disrupted FC, and microstructural damage within the prefrontal cortex, hippocampus, and visual networks.^88^ These neural disruptions correlate with cognitive and emotional impairments, underscoring TED’s systemic neurological impact.

Structural and functional brain network disruptions (Table 4) in TED vary by disease phase and subtype. Inactive TED patients retain small-world organization in structural networks, reflecting preserved global integration.^74^ In contrast, active TED has been characterized by reduced global efficiency and local efficiency, reflecting impaired integration and segregation of information.^75^ White matter networks in TED have been reported to exhibit reduced modularity and small-worldness, with decreased betweenness centrality in the corpus callosum, implicating disorganized inter-module communication critical for cognitive-visual integration.^78^ Notably, patients with DON, exhibit further alterations of static functional networks, marked by decreased small-worldness, lower clustering coefficients, and prolonged characteristic path length, suggesting increased network instability or reconfiguration.^76^

Nodal disruptions in TED localize to emotion- and vision-related circuits. Active TED patients demonstrate reduced nodal efficiency in the orbital superior frontal gyrus, hippocampus, and amygdala, correlating with anxiety and depression severity,^75^ while DON patients show hypo-connectivity in the orbitofrontal and visual networks.^76^ Conversely, compensatory hyperconnectivity emerges in the left cuneus and intraparietal sulcus, potentially offsetting visual deficits.^74^^,^^77^

Clinically, network metrics have been reported to correlate with functional outcomes. Researches showed that prefrontal nodal efficiency is inversely associated with psychiatric symptoms,^75^ while dynamic network variability distinguishes DON from non-DON in research cohorts, with high classification performance.^76^ These findings suggest that graph-theoretical approaches may support disease stratification and longitudinal monitoring in TED. In summary, TED’s autoimmune pathophysiology likely extends to the CNS, contributing to both ocular and extra-ocular symptoms. Brain imaging provides a promising research avenue to investigate TED’s systemic impact and opens new directions for studying autoimmune brain-eye interactions.

### T1DM

T1DM is a chronic autoimmune and metabolic disease, typically manifesting in childhood or adolescence, characterized by an absolute deficiency in insulin due to autoimmune destruction of pancreatic β-cells.^89^ Prolonged hyperglycemia in T1DM patients often leads to microvascular complications, including diabetic retinopathy (DR), with proliferative diabetic retinopathy (PDR) being a common advanced manifestation.^90^ Additionally, patients frequently experience cognitive impairments, such as reduced information processing speed and diminished attention.^91^^,^^92^ Neuroimaging studies reveal widespread structural and functional brain alterations, including reduced fractional anisotropy, mean diffusivity, and axial diffusivity across cortical and subcortical regions, reflecting microstructural damage and disorganization in white matter integrity.^93^

T1DM is associated with disruptions in brain network topology, retaining small-world properties structurally but exhibiting reduced global efficiency, clustering coefficient, and local efficiency, with increased characteristic path length versus controls^79^^,^^80^ (Figure 2; Table 4). These deficits are exacerbated in patients with PDR, who display randomized global gray matter topology and region-specific reductions in local clustering coefficients, particularly within the middle frontal and post-central cortices.^80^ Such alterations correlate with cognitive decline, explaining up to 20% of variance in performance, and are associated with reduced fractional anisotropy in white matter tracts, implicating microstructural damage in network dysfunction.^80^

Functionally, T1DM patients demonstrate diminished eigenvector and degree centrality in the bilateral thalamus and dorsal striatum, with the most pronounced reductions in T1DM PDR cohorts, while non-PDR patients show compensatory increases in the lateral occipital cortex and right cuneus, suggesting adaptive visual-associative reorganization. This compensatory hierarchy declines with disease progression, aligning with disrupted connectivity in SMN, auditory, and language resting-state networks.^81^ These findings suggest that T1DM drives prefrontal hub loss, executive-limbic disintegration, and ineffective compensation, linking metabolic dysregulation to cognitive decline.

### IBD

IBD, encompassing Crohn’s disease (CD) and ulcerative colitis (UC), is a chronic inflammatory disorder of the gastrointestinal tract characterized by abdominal pain, altered bowel habits, and rectal bleeding.^94^^,^^95^ It is characterized by aberrant innate and adaptive immune responses that trigger persistent intestinal inflammation.^96^ Beyond gastrointestinal symptoms, over 30% of IBD patients experience comorbid psychological disorders such as anxiety and depression, underscoring the disease’s systemic and brain-gut axis implications.^97^

Resting-state fMRI studies (Figure 2; Table 4) reveal disrupted global and local FC in CD patients, particularly in subcortical, cognitive control, SMN, and DMN. These disruptions have been found to correlate with clinical features: reduced putamen connectivity associates with longer disease duration, while anxiety/depression symptoms correspond to altered nodal efficiency in the anterior cingulate cortex and medial prefrontal cortex.^82^ MEG further identifies increased betweenness centrality in the left hippocampus (gamma band) in IBD patients, indicating enhanced information flow through this region. Notably, these topological changes are independent of IBD subtype (UC vs. CD), suggesting inflammation itself drives brain reorganization rather than disease-specific mechanisms.^83^ These findings highlight how chronic intestinal inflammation disrupts brain networks involved in pain processing, emotion regulation, and cognitive control, advancing our understanding of the gut-brain axis in IBD pathophysiology.

## Discussion

Autoimmune diseases, encompassing both organ-specific and systemic disorders, impact brain networks through direct and indirect mechanisms. Direct effects may arise from autoantibodies that trigger chronic inflammation, which may lead to structural alterations in brain networks. For instance, elevated levels of macrophage colony-stimulating factors in the central and peripheral nervous systems can activate microglial cells, promoting neuroinflammation and disrupting neural communication.^98^^,^^99^^,^^100^ Indirectly, systemic changes from these diseases may affect specific brain regions. For example, structural and microvascular damage in the optic nerve of patients with NMOSD and TED influences neural connectivity, creating a bidirectional effect where changes in brain connectivity also affect optic nerve structure and function.^56^ In T1DM, hyperglycemia damages the retina and peripheral nerves, potentially leading to structural brain network changes as the brain adapts to compensate for peripheral damage.^15^

Across autoimmune diseases, heterogeneous network alterations converge on several shared organizational principles when considered along disease state and network stress dimensions rather than diagnostic categories. During periods of active immune-mediated CNS involvement, multiple disorders exhibit reduced global efficiency and increased characteristic path length,^59^^,^^68^^,^^75^ suggesting impaired long-range information integration. Longer disease duration or sustained immune exposure is more frequently associated with modular reorganization and selective hub vulnerability rather than diffuse network randomization.^45^^,^^69^^,^^76^ Additionally, several studies report early or region-specific increases in connectivity^32^^,^^39^ likely reflecting compensatory or maladaptive plasticity that may precede subsequent network destabilization.

**Figure 3:**
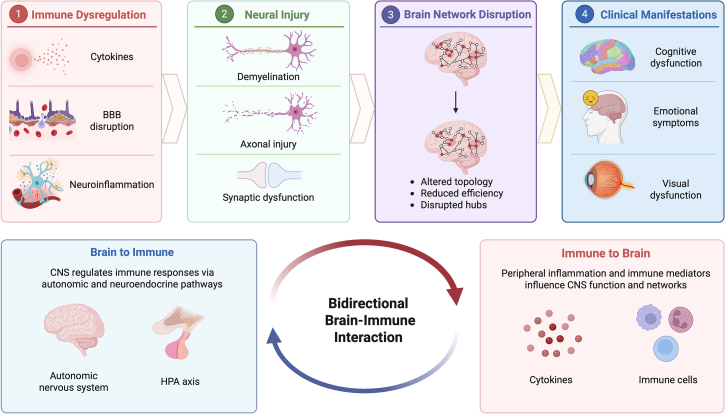
Bidirectional immune-brain interactions in autoimmune diseases Peripheral immune activation and pro-inflammatory cytokines promote blood-brain barrier dysfunction, neuroinflammation, glial activation, and altered brain network connectivity, leading to cognitive impairment and sickness behavior. In parallel, the brain modulates peripheral immune activity through the HPA axis and autonomic pathways, highlighting a dynamic feedback loop between immune dysregulation and brain network alterations. HPA, hypothalamic-pituitary-adrenal.

These patterns are broadly consistent with a bidirectional immune-brain interaction model (Figure 3); however, the directionality of effects requires cautious interpretation. Current evidence is predominantly derived from cross-sectional, correlational studies, and causal inferences attributing network alterations directly to immune attack therefore remain tentative. A plausible interpretation is that peripheral immune activation may contribute to network topological changes via mechanisms such as neuroinflammation, blood-brain barrier dysfunction, and glial activation.^101^ Peripheral inflammation can trigger long-lasting epigenetic reprogramming in microglia, leading to either a pro-inflammatory or tolerogenic phenotype that persists for months and influences synaptic function and connectivity.^102^ Circulating cytokines such as interleukin-1β and tumor necrosis factor-α further contribute to cognitive impairment and altered network dynamics by inducing sickness behaviors and disrupting neuronal signaling.^103^ At the same time, the brain modulates peripheral immune responses through autonomic pathways, and specific brain regions, such as the insular cortex can store and retrieve immune information, actively shaping immune activity.^104^ These findings suggest that immune dysregulation in autoimmunity may both result from and contribute to altered brain network architecture, helping explain the neuropsychiatric and cognitive symptoms commonly observed in these conditions.

Importantly, not all studies report uniform network degradation in autoimmune diseases. Several investigations describe preserved global topology, increased local efficiency, or region-specific hyperconnectivity.^32^^,^^36^^,^^39^ Such findings may reflect compensatory or adaptive network reorganization rather than methodological inconsistency. Null findings and mixed directions of change further highlight the heterogeneity of network responses across disease stage, subtype, and imaging modality.

Given that autoimmune diseases often involve multi-organ dysfunction and immune system dysregulation, these conditions can impair brain function and potentially cause psychological issues and cognitive decline.^105^ Research on brain network function and structure further supports these observations. Numerous studies indicate significant correlations between specific network metrics and declines in cognitive and memory functions,^73^^,^^74^^,^^80^ suggesting that changes in brain network structure may underlie cognitive and memory impairment in autoimmune diseases. Additionally, variations in brain network metrics have been associated with certain immune markers.^73^ From a translational perspective, graph theory-based brain network analysis provides a quantitative framework for characterizing system-level brain alterations associated with autoimmune diseases. At present, these network metrics should be viewed as biomarkers of disease burden, cognitive vulnerability, and longitudinal trajectory rather than direct guides for therapeutic intervention. Although advances in neuroimaging have outpaced the development of precision immunotherapies, network-level measures may still support patient stratification,^72^^,^^76^ risk assessment,^31^^,^^49^ and monitoring of disease evolution, or treatment response.^46^ Importantly, identifying early or compensatory network reorganization may inform hypothesis-driven clinical studies and improve understanding of how immune-mediated processes influence brain function over time, without implying immediate clinical actionability.

Despite progress, current research on brain network alterations in autoimmune diseases faces several key limitations. Methodologically, most existing studies are cross-sectional, relying on single scans to evaluate brain network metrics, which limits the ability to predict disease progression. Longitudinal studies, like the work of Koubiyr et al. in early stage MS, are rare but crucial to understanding how autoimmune diseases affect brain networks over time.^31^ While fMRI is the predominant modality in current research, other technologies such as positron emission tomography (PET), especially when combined with molecular probes targeting inflammation or glial activation remain underutilized. Integrating molecular imaging can enhance our understanding of pathophysiological changes at the cellular level and complement the macroscopic connectivity patterns revealed by fMRI. Additionally, the lack of standardized protocols for graph theory analysis introduces variability, particularly in threshold selection and parcellation schemes, which directly impact metric reproducibility. Brain network metrics are also affected by segmentation schemes, with no consensus on the optimal approach for capturing individual differences in functional or anatomical connectivity. Imaging modality differences, such as resolution disparities across structural and functional scans, also impact the precision and comparability of network metrics.

Disease-related limitations include a narrow focus on select conditions, while this review synthesizes findings from MS, NMOSD, SLE, TED, T1DM, and IBD, other prevalent autoimmune diseases such as RA, Sjögren’s syndrome, and myasthenia gravis remain underrepresented in brain network studies. This limited disease scope restricts the generalizability of current findings and hampers the identification of shared neuroimmune mechanisms across autoimmune conditions. Furthermore, brain network analyses face challenges like patient heterogeneity, as autoimmune diseases are highly variable in presentation and severity, which could influence network findings. Critically, few studies directly correlate autoimmune biomarkers such as autoantibodies, with network-level findings. Thus, the relative contributions of autoimmune-specific mechanisms versus secondary systemic effects to brain network disruption remain unclear. Incorporating data on anti-N-methyl-d-aspartate receptor (anti-NMDAR), anti–Sjögren's-syndrome-related antigen A and B autoantibodies (anti-SSA/SSB), or antiphospholipid antibodies could provide critical insights into how specific components of autoimmunity relate to neural network disruptions. Multivariate analyses controlling for systemic variables could clarify whether network alterations are primarily immune-driven or secondary to broader metabolic or endocrine dysfunction. Connectomic findings in autoimmune diseases are highly sensitive to analytic choices. Proportional thresholding may result in the inclusion of spurious connections in datasets,^106^ while head motion is associate with decreased functional coupling in the default and frontoparietal control networks.^107^ dMRI networks are further limited by tractography false positives and algorithmic variability,^108^ particularly under inflammatory microstructural changes. Network metrics also depend on parcellation scheme and scanner or protocol heterogeneity, often without formal harmonization. Consequently, reported network alterations should be interpreted as method-dependent tendencies rather than invariant disease signatures, emphasizing the need for standardized and transparent analytic pipelines.

Addressing these limitations will require a multifaceted research strategy. Most available studies are cross-sectional, involve modest sample sizes, and employ heterogeneous acquisition and preprocessing pipelines. Therefore, reported network alterations should be interpreted as group-level tendencies rather than uniform disease traits. Future studies should prioritize longitudinal designs, expand to include a broader range of autoimmune diseases, and strive for methodological standardization in graph theory-based analysis. Multimodal imaging approaches that integrate structural, functional and molecular data, could offer a more comprehensive view of brain network alterations in autoimmune diseases. Artificial intelligence and machine learning are promising tools to analyze complex network patterns, potentially improving diagnostic accuracy and predictive modeling for disease progression. Large language and foundation models are increasingly applied in radiology, particularly for multimodal disease classification and prognostic prediction.^109^ Through these advanced techniques and standardized approaches, the field can move toward a clearer understanding of how autoimmune diseases impact brain networks, paving the way for more accurate diagnoses and targeted interventions. Accordingly, the present synthesis should be viewed as a conceptual framework rather than a definitive model, intended to guide hypothesis-driven and longitudinal studies rather than establish causal mechanisms.

## Conclusion

Brain network research in autoimmune diseases, including MS, SLE, NMOSD, TED, T1DM, and IBD, has consistently reported significant reductions in global and local efficiency despite preserved small-world properties. Regional connectivity alterations vary by disease, suggesting distinct mechanisms that may contribute to cognitive impairment. The correlation between network metrics and clinical indicators suggests potential applications for these metrics as biomarkers to monitor disease progression and aid in diagnostics. However, current findings should be interpreted cautiously given the methodological heterogeneity and predominance of cross-sectional studies. Future research should focus on larger sample sizes, standardized methods, and longitudinal designs to enhance the reliability and clinical relevance of findings.

## Data availability

No new datasets were generated or analyzed in this study. All extracted information supporting the findings of this review is included within the article and its tables.

## Acknowledgments

This work was supported by the 10.13039/501100012166National Key R&D Program of China (2024YFB4710200, 2024YFB4710205); the 10.13039/501100001809National Natural Science Foundation of China (82388101); the Science and Technology Commission of Shanghai (20DZ2270800); Shanghai Key Clinical Specialty, Shanghai Eye Disease Research Center (2022ZZ01003); The Research Center for Eye Disease and Visual Rehabilitation, and the Key Project of Yuanshen Rehabilitation Institute Shanghai Jiao Tong University School of Medicine (yskf1-24-0926-2; yskf2-24-0926-2).

## Author contributions

X.N. Lee, conceptualization, visualization, writing – original draft; H. Zhang, conceptualization, methodology, writing – original draft; H. Zhou, project administration, supervision, funding acquisition, writing – review and editing.

## Declaration of interests

The authors have no competing interests to declare that are relevant to the content of this article.
